# High dose androgen suppresses natural killer cytotoxicity of castration-resistant prostate cancer cells via altering AR/circFKBP5/miRNA-513a-5p/PD-L1 signals

**DOI:** 10.1038/s41419-022-04956-w

**Published:** 2022-08-29

**Authors:** Min Tang, Yin Sun, Chi-Ping Huang, Lei Chen, Bianjiang Liu, Bosen You, Zengjun Wang, Chawnshang Chang

**Affiliations:** 1 Department of Urology, The First Affiliated Hospital of Nanjing Medical University, Nanjing, Jiangsu 210000 China; 2 George Whipple Lab for Cancer Research, Departments of Pathology, Urology, Radiation Oncology and The Wilmot Cancer Institute, University of Rochester Medical Center, Rochester, 14642 NY USA; 3 Department of Urology, China Medical University/Hospital, Taichung, 404 Taiwan; 4 Department of Urology, The Second Affiliated Hospital of Anhui Medical University, Hefei, Anhui 230000 China

**Keywords:** Cell signalling, Prostate cancer

## Abstract

Most advanced prostate cancer (PCa) patients initially respond well to androgen deprivation therapy, but almost all eventually develop castration-resistant prostate cancer (CRPC). Early studies indicated the bipolar androgen therapy via a cycling of high dose and low dose of androgen to suppress PCa growth might be effective in a select patient population. The detailed mechanisms, however, remain unclear. Here we found the capacity of natural killer (NK) cells to suppress the CRPC cells could be suppressed by a high dose of dihydrotestosterone (DHT). Mechanism dissection indicates that transactivated AR can increase circularRNA-FKBP5 (circFKBP5) expression, which could sponge/inhibit miR-513a-5p that suppresses the PD-L1 expression via direct binding to its 3ʹUTR to negatively impact immune surveillance from NK cells. Preclinical data from in vitro cell lines and an in vivo mouse model indicate that targeting PD-L1 with sh-RNA or anti-PD-L1 antibody can enhance the high dose DHT effect to better suppress CRPC cell growth. These findings may help us to develop novel therapies via combination of high dose androgen with PD-1/PD-L1 checkpoint inhibitors to better suppress CRPC progression.

## Introduction

Androgen deprivation therapy (ADT) has remained the primary treatment for advanced prostate cancer (PCa) over the 70 years since 1941 when Dr. Charles Huggins discovered the significant palliative benefit of ADT by surgical castration or estrogen therapy in patients with symptomatic, advanced PCa [1, 2]. However, PCa in nearly all patients developed to the castration-resistant prostate cancer (CRPC) stage due to sustained androgen receptor (AR) signaling in response to chronic exposure to low testosterone through different mechanisms [3–5]. Enzalutamide (Enz), a potent antiandrogen, has played a significant role in fighting CRPC or hormone-sensitive prostate cancer (HSPC) [6, 7]. Unfortunately, most patients develop Enz-resistance quickly [8] with a poor prognosis and quality-of-life as well as limited therapeutic choices.

Bipolar androgen therapy (BAT) has been considered a novel therapeutic option to effectively inhibit CRPC via a cycling of supraphysiological and near-castrate testosterone levels, achieved by intermittent testosterone injections and continuous near castration therapy [2, 9]. BAT can not only improve the quality-of-life for CRPC patients, but also restore sensitivity to ADT [2]. However, the exact mechanisms mediating the effects of high dose androgen treatment are still not clear. Several hypotheses for the paradoxical efficacy of BAT have been described. One of them suggested that the overstabilization of high dose androgen-activated AR might prevent its degradation and inhibit licensing of DNA replication, ultimately leading to cell death [10]. Another hypothesis suggested that high dose dihydrotestosterone (DHT) may induce lethal double-strand DNA breaks (DSB) via recruiting AR and topoisomerase IIβ (TOP2B) to androgen response elements (AREs) in CRPC cells [11, 12]. Understanding detailed mechanisms underlying its efficacy could help us to develop new targeted therapies to enhance its efficacy.

Recent studies revealed that, apart from prostate stromal cells, AR is also expressed in various types of immune cells, exerting significant influences on both innate and adaptive immune regulations [13–15]. It was reported that medical ADT, involving AR antagonists, could suppress the adaptive immune responses via perturbing initial T cell priming [16]. Additionally, it was found that natural killer (NK) cells can preferentially target androgen-dependent prostate cancer stem-like cells via the TRAIL/DR5 pathway [17]. These findings suggest that a high dose androgen might impact the immune functions through its intended target in the PCa as well as directly on immune cells.

We report here that a high dose androgen through AR can inhibit NK cell cytotoxicity towards EnzS1-C4-2 and EnzR1-C4-2 cells via the circFKBP5/miR-513a-5p/PD-L1 pathway. Therefore, targeting PD-L1 with anti-PD-L1 antibody or knocking down PD-L1 expression could increase the inhibitory effect of high dose androgen treatment to better suppress the growth of CRPC cells, through enhancing NK cytotoxicity.

## Materials and methods

### Cell culture

The human PCa cell line C4-2 (EnzS1-C4-2) was obtained from American Type Culture Collection (ATCC, Manassas, VA). EnzR1-C4-2 cells were generated by culturing EnzS1-C4-2 cells under increasing Enz concentrations from 10 μM to 40 μM (every 20 days) for three months, and then maintained in media with 10 µM Enz. Both EnzS1-C4-2 and EnzR1-C4-2 cells were cultured in RPMI-1640 (without phenol red) supplied with 2 nM DHT, 10% fetal bovine serum (FBS) and 1% Penicillin-Streptomycin. The human normal cells HEK-293T (ATCC, Manassas, VA) were cultured in DMEM media. NK-92MI cells (ATCC, Manassas, VA) were maintained in α-MEM (Invitrogen, Grand Island, NY) with 0.2 mM inositol, 0.1 mM 2-mercaptoethanol, 0.02 mM folic acid, FBS to a final concentration of 12.5% and horse serum to a final concentration of 12.5% based on ATCC guidelines. All cell lines were cultured in a 5%(v/v) CO_2_ humidified incubator at 37 °C.

### Plasmids and lentivirus packaging

The plasmids pLKO.1-AR, pLKO.1-FKBP5, pLKO.1-circ76151, pLKO.1-circ127664, pLKO.1-miR-513a-5p, pLKO.1-PD-L1, pWPI-circ76151-wild type, pWPI-circ76151-mutant, psiCHECK2-wild type, and psiCHECK2-mutant were packaged with psPAX2 packaging plasmid and pMD2.G envelope plasmid, then transfected into HEK-293T cells with the standard calcium chloride transfection method for 48 h. The lentivirus suspension was collected and used immediately or frozen at −80 °C for later use.

### RNA extraction and quantitative real-time RT-PCR (qRT-PCR) analysis

Total RNA was extracted and isolated with Trizol reagent (Invitrogen), and then 1 μg of total RNA was used for reverse transcription into cDNA with Superscript III transcriptase (Invitrogen). For the miRNA reverse transcription, 1 μg total RNA was used for poly A addition reaction with the conditions as follows: 1 mM ATP in 1 × RT buffer up to 10 μl volume at 37 °C for 20 min. The mRNA level determination was performed using a Bio-Rad CFX96 system with the conditions as follows: 95 °C, 2 min; 95 °C,15 s; 60 °C, 45 s for 45 cycles. U6 and 18 S were used as an internal standard control for miRNA and mRNA detection, respectively. Each sample was replicated three times and data was analyzed by comparing Ct values.

### Western blot analysis

Cells were lysed in RIPA buffer and proteins (30 µg) were separated on 8–10% SDS/PAGE gel and then transferred onto PVDF membranes (Millipore, Billerica, MA). After blocking membranes, they were incubated with appropriate dilutions of specific primary antibodies, and then blots were incubated with HRP-conjugated secondary antibodies and visualized using the ECL system (Thermo Fisher Scientific, Rochester, NY).

### Flow cytometry

0.5–1 × 10^6^ cultured cells were collected with cell scrapers and resuspended in 100 µL incubation buffer (0.25 g bovine serum albumin in 50 mL 1 × PBS) with 20 µL of FITC-conjugated mouse anti-human primary PD-L1 monoclonal antibody (BD Pharmingen, P/N 558065) for 1 h at room temperature. Cells were rinsed twice in incubation buffer and finally resuspended in 0.45 mL ice cold 1 × PBS and strained into polystyrene flow cytometry tubes. Flow Cytometry was conducted using the CytoFLEX S Flow Cytometer–Beckman Coulter and each experiment was conducted in triplicate.

### MTT assay

The cell viability was detected via MTT assay. Two CRPC cell lines, EnzS1-C4-2 and EnzR1-C4-2, were treated with Ethanol (EtOH) or high dose DHT (50 nM) for 48 h. Then the treated cells were plated at a density of 1.5 × 10^4^ per well on 24-well plates. After seeding overnight, NK-92MI cells were added at multiple E (Effector cell):T (Target cell) ratios (1:1, 2:1, and 5:1) and co-cultured with the cells. After co-culture for 36 h, the NK cells were removed from the culture wells by washing twice with 1 × PBS. Then, 10 μl MTT(5 mg/ml) reagent was added into each well and incubated for 1 h at 37 °C. After discarding the supernatant, 100 μl DMSO (Amresco Inc., Boise, Idaho) was added into each well to dissolve the crystals and the OD value was determined at 570 nm absorbance. High dose DHT treatment groups were compared to non-treatment group.

### LDH assay

NK cells cytotoxicity against CRPC cells was analyzed using a lactate dehydrogenase (LDH) assay as described by Simon Kaja [18]. Briefly, EnzS1-C4-2 or EnzR1-C4-2 cells that have been treated with high dose DHT for 48 h were seeded into 96-well plates (the sample plate) at a density of 7500 cells/well. Then cells were exposed to NK cells in different E:T ratios (1:1, 2:1, 5:1) after seeding overnight. Following 24 h exposure to NK cells, 50 μl of cell culture media from this mixed culture was transferred into a new clear, untreated 96-well plate (the assay plate). Then 50 μl Assay Buffer (2 mM iodonitrotetrazolium chloride (INT), 3.2 mM β-nicotinamide adenine dinucleotide sodium salt (NAD), 160 mM lithium lactate, 15 μM 1-methoxyphenazine methosulfate (MPMS) in 0.2 M Tris-HCl buffer, pH 8.2) was added. Plates were incubated at room temperature in the dark for 1 hr. The reaction was stopped by addition of 50 μl 1 M acetic acid. LDH release was quantified by measuring absorbance at 490 nm (A490) using a Synergy H1 plate reader (Biotek, Winooski, VT). Data were exported to Microsoft Excel for processing, normalized to the control condition, and analyzed in Prism 7.0 (Graphpad, La Jolla, CA).

Stock solutions for the LDH Assay Buffer were prepared in advance and consisted of Buffer A (2×; 4 mM INT in 0.2 M Tris-HCl buffer, pH 8.2), Buffer B (2×; 6.4 mM NAD, 320 mM lithium lactate in 0.2 M Tris-HCl buffer, pH 8.2), and MPMS supplement (10,000×; 150 mM MPMS in 0.2 M Tris-HCl buffer, pH 8.2).

### RNA immunoprecipitation (RIP)

We lysed the cells in ice-cold lysis buffer supplemented with RNase inhibitor. After centrifugation, 10 μg of the supernatant was cleared by protein A/G beads for 1 h and incubated with Agonaute-2 (AGO2) antibody overnight at 4 °C. Then the beads were added to the antibody-lysate mixture and incubated for another 2 h. The RNA/antibody complex was washed four times by RIPA buffer supplemented with RNase inhibitor, protease inhibitor cocktail. The RNA was extracted using Trizol (Invitrogen) according to the manufacturer’s protocol and subjected to qRT-PCR analysis.

### PD-L1-miRNA Pull-down assay

The cell samples were collected and lysed in cold RNA immunoprecipitation (RIP) lysis buffer mixed with RNase inhibitor (M0307S, NEB; Ipswich, MA, USA). After centrifugation, the supernatant was collected into a new Eppendorf and was mixed with protein A/G beads for 2 h at 4 °C. Then, 10 μl biotin-labelled anti-sense oligos against PD-L1 was added into the cell lysate mixture and rotated overnight at 4 °C. The next day, 10 μl streptavidin agarose beads were added into the mixture and rotation continued for 2 h. After centrifuged at 3000 rpm for 10 min, the beads were washed 10 times with cell lysis buffer. Finally, the total RNA was extracted with Trizol and qRT-PCR assay was performed as previously described.

### Luciferase reporter assay

The PD-L1 3’UTR with wild type or mutant miRNA-responsive elements was cloned into the psiCHECK-2 vector (Promega, Madison, WI) downstream of the Renilla luciferase ORF. EnzS1-C4-2 and EnzR1-C4-2 cells, which have already been transfected by pLKO-miR-513a-5p plasmid, were plated in 24-well plates and the plasmids were transfected with Lipofectamine 3000 transfection reagent (Invitrogen, Carlsbad, CA) according to the manufacturer’s instructions. Luciferase activity was measured 48 h after transfection by Dual-Luciferase Assay (Promega) according to the manufacturer’s manual.

### In vivo studies

All the animal experiments were performed in accordance with the guidelines for the care and use of laboratory animals and were approved by the Medical Center Animal Care and Use Committee of Nanjing Medical University. First, 5 × 10^6^ EnzR1-C4-2 cells were transfected by pLKO.1 or pLKO.1-sh-PD-L1 and mixed with matrigel (1:1) for transplantation subcutaneously into six weeks old male Balb/c nude mice purchased from Cavens (Changzhou, China). When tumors develop in ~2 weeks each set of mice were randomly divided into four treatment groups as follows: 1: pLKO+EtOH; 2: pLKO+Testosterone; 3: sh-PD-L1 + EtOH; and 4: sh-PD-L1 + Testosterone. Enz was given to all mice through intraperitoneal injection (10 mg/kg/week, twice weekly). BAT treatment was conducted by injected administration of Testosterone (200 μg/kg, twice weekly) or EtOH during the 3rd/5th/7th weeks. Tumors were measured weekly by caliper. Mice were sacrificed after 8 weeks, tumors were removed to determine tumor volume and for IHC studies.

### Immunohistochemistry (IHC) staining

IHC was performed on the samples from mouse tumors. Briefly, the samples were fixed in 4% neutral buffered paraformaldehyde for 18 h, then embedded in paraffin and cut into 4 μm slices. After deparaffnization, hydration, antigen retrieval, and blocking, these sections were incubated with corresponding primary antibodies, incubated with biotinylated secondary antibodies (Vector Laboratories, Burlingame, CA, USA), and then visualized by VECTASTAIN ABC peroxidase system and 3, 3’-diaminobenzidine (DAB) kit (Vector Laboratories, Burlingame, CA, USA). The slides were reviewed separately by two experienced pathologists. Average optical density of staining was calculated using Image J software.

### Statistics

All experiments were performed least three times with data points in triplicate. All statistical analyses were carried out with SPSS 19.0 (SPSS Inc, Chicago, IL). The data values were presented as the mean ± SD. Differences in mean values between two groups were analyzed by two-tailed Student’s t test and the mean values of more than two groups were compared with one way ANOVA. *p* ≤ 0.05 was considered statistically significant.

## Results

### A high dose DHT through AR can suppress the cytotoxicity of NK cells to kill CRPC cells

**Fig. 1:**
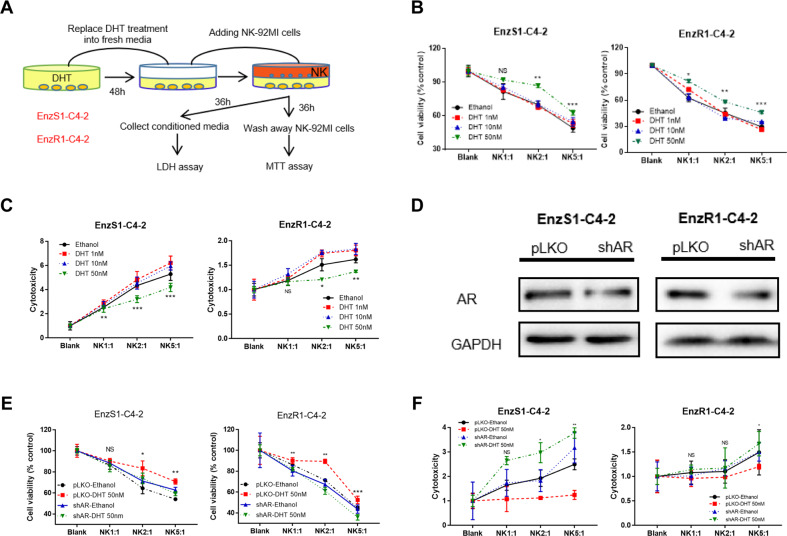
A high dose DHT treatment through AR can suppress the cytotoxicity of NK cells towards CRPC cells. **A** Cartoon for NK cells cytotoxicity test procedure by MTT and LDH assay. **B**, **C** EnzS1-C4-2 (left panel) and EnzR1-C4-2 (right panel) were treated with different dosages of DHT (1 nM, 10 nM, and 50 nM) for 48 h, then NK-92MI cells were added with multiple E:T ratios (1:1, 2:1, and 5:1) for 36 h before performing MTT assay (**B**) and LDH assay (**C**). **D** Western blot shows AR expression after knocking down with shAR in both cell lines. **E**, **F** Before treating EnzS1-C4-2 and EnzR1-C4-2 cells with 50 nM DHT, AR expression was knocked down. After treatment, MTT (**E**) and LDH assays (**F**) were performed separately using NK92-MI cells to target CRPC cells. *p < 0.05, **p < 0.01, ***p < 0.001, NS Not Significant.

EnzS1-C4-2 and EnzR1-C4-2 cells were treated with different doses of DHT (1 nM, 10 nM and 50 nM) for 48 h. Results from the MTT assay (detailed procedure described in Fig. 1A) showed that adding NK cells with multiple E:T ratios (1:1, 2:1 and 5:1) to high dose DHT (50 nM)-treated CRPC cells resulted in impaired killing efficacy of NK cells (Fig. 1B). To directly measure the leakiness of plasma membrane caused by the pore forming cytolytic perforin and granzymes from the NK cells, we measured the lactate dehydrogenase activity in the media after NK cell co-culture (detailed procedure described in Fig. 1A). The results revealed that adding NK cells to these 50 nM DHT-treated CRPC cells resulted in reduced efficacy of NK cytotoxicity (Fig. 1C). These complementing results indicated that a high dose DHT could reduce the immunological efficacy of NK cells towards CRPC cells. In comparison, NK cytotoxicity was not weakened by treatment with lower doses (1 nM and 10 nM) of DHT (Fig. 1B, C).

As DHT can impact cells in an AR-independent manner such as conversion to other steroid hormones, we also examined whether AR protein was directly involved in mediating this DHT effect. We knocked down the expression of AR in EnzS1-C4-2 and EnzR1-C4-2 cells (Fig. 1D) by AR-shRNA followed by a high dose DHT treatment. The results of MTT **(**Fig. 1E**)** and LDH (Fig. 1F**)** assays indicated that, compared with the control group, the inhibition effect of NK cytotoxicity towards CRPC cells was reversed in the cells with a lower AR expression.

Together, results from Fig. 1A–F suggest high dose DHT through AR can suppress the cytotoxicity of NK cells to kill CRPC cells.

### A high dose DHT/AR suppresses NK cells immunotherapy efficacy to kill CRPC cells via up-regulating PD-L1 expression in CRPC cells

**Fig. 2:**
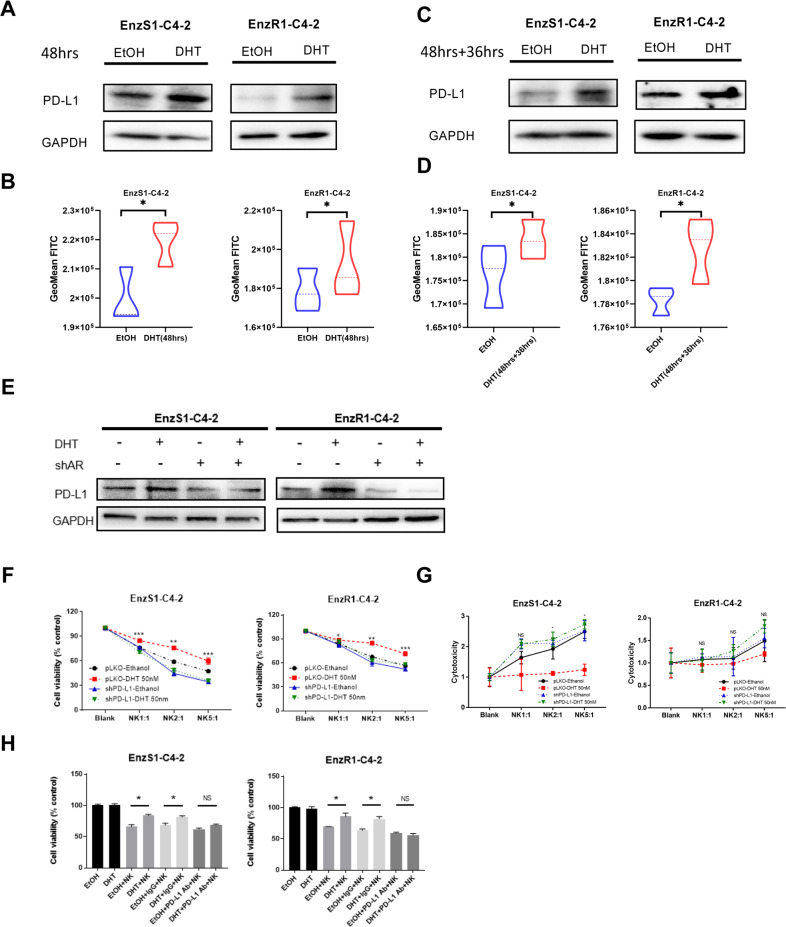
A high dose DHT/AR suppresses NK cells immunotherapy efficacy towards CRPC cells via up-regulating PD-L1 expression in CRPC cells. **A**, **B** After treating EnzS1-C4-2 (left panel) and EnzR1-C4-2 (right panel) cell lines with 50 nM DHT for 48 h, the protein was extracted to run western blot (**A**) to test PD-L1 protein expression and Flow cytometry (**B**) showed the change in surface expression of PD-L1. GeoMean FITC standed for relative expression of PD-L1 **C**, **D** After treated with 50 nM DHT for 48 h, the CRPC cells were allowed to grow in fresh media for another 36 h. Then total PD-L1 protein was measured with western blot (**C**) and surface PD-L1 protein was detected with flow cytometry (**D**). **E** Full length AR was inhibited in EnzS1-C4-2 (left panel) and EnzR1-C4-2 (right panel) cells, cells were treated with 50 nM DHT for 48 h, then western blot was performed to test PD-L1 changes compared with scr group. **F**, **G** Before treating EnzS1-C4-2 and EnzR1-C4-2 cells with 50 nM DHT, PD-L1 expression was knocked down. After treatment, MTT (**F**) and LDH assay **G** were performed separately using NK92-MI cells to target CRPC cells. **H** After treating EnzS1-C4-2 and EnzR1-C4-2 cells with 50 nM DHT for 48 h, PD-L1 neutralizing antibody or IgG control antibody, as well as NK92-MI cells, were added for 36 h, then MTT assay was performed to evaluate the NK cell cytotoxicity. *p < 0.05, **p < 0.01, ***p < 0.001, NS Not Significant.

It was reported that PD-L1 expression in cancer cells could lead to reduced NK cell responses via altering the PD-1/PD-L1 inhibitory axis [19]. To test whether PD-L1 might be mediating this effect to impact CRPC cell viability, we directly measured the expression of PD-L1 in response to a high dose DHT in EnzS1-C4-2 and EnzR1-C4-2 cells. We found a higher expression of PD-L1 protein after treatment with a high dose DHT for 48 h (Fig. 2A, B). Indeed, this increased PD-L1 expression persisted at least 36 h in the absence of DHT after 48 h treatment with a high dose DHT, likely impacting the NK cells killing as described earlier (Fig. 2C, D). Furthermore, when AR expression was knocked down, the increase of PD-L1 protein resulting from high dose DHT was suppressed (Fig. 2E). These results support the notion that a high dose DHT through AR could up-regulate PD-L1 protein expression in CRPC cells.

To determine whether PD-L1 plays an essential role in the inhibition of NK cells killing capacity, we suppressed PD-L1 in EnzS1-C4-2 and EnzR1-C4-2 cells and then treated with high dose DHT. Results with MTT **(**Fig. 2F**)** and LDH **(**Fig. 2G**)** assays showed that shRNA-mediated reduction of PD-L1 could prevent the decrease of NK cell killing capacity induced by high dose DHT. In addition, we directly incubated EnzS1-C4-2 and EnzR1-C4-2 cells with either 1 μg of PD-L1 rabbit polyclonal antibody (Abclonal, Woburn, MA) or rabbit IgG control antibody under high dose DHT. The results of MTT assay showed that blocking PD-L1 with anti-PD-L1 antibody resulted in partial reverse of NK cells cytotoxicity (Fig. 2H).

Taken together, these results suggested that a high dose DHT through AR can reduce the NK cell killing capacity by up-regulation of PD-L1 expression in CRPC cells. It also suggests that BAT with high doses of androgen might be enhanced with targeting PD-L1 expression.

### Mechanism dissection of how a high dose DHT/AR can increase the PD-L1 expression: via suppressing miRNAs function

**Fig. 3:**
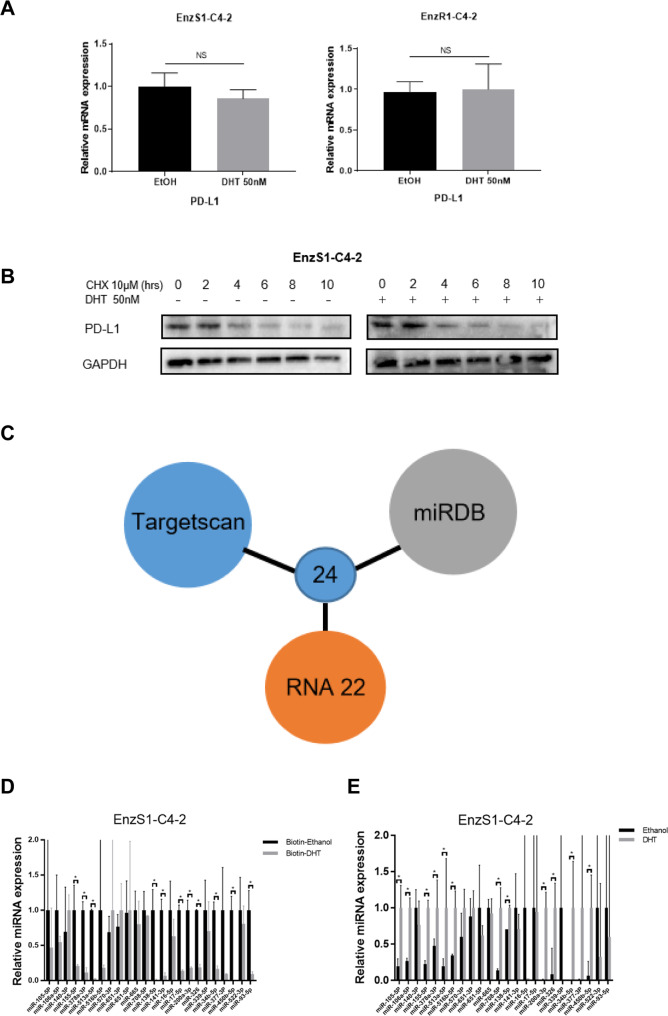
A high dose DHT/AR can increase the PD-L1 expression via suppressing miRNAs function. **A** qRT-PCR was performed to test the PD-L1 mRNA expression in EnzS1-C4-2 (left panel) and EnzR1-C4-2 (right panel) after high dose DHT treatment for 48 h. **B** PD-L1 protein stability after 50 nM DHT treatment at 0, 2, 4, 6, 8, and 10 h. **C** Venn diagram for prediction of candidate miRNAs targeting PD-L1 using 3 miRNA related websites (Targetscan, miRDB and RNA 22). **D** miR-155-5p, miR-378a-3p, miR-513a-5p, miR-138-5p, miR326, miR-141-3p, miR-17-5p, miR-200a-3p, miR-34b-5p, miR-450b-5p, and miR-93-5p, not the other 13 miRNAs, were decreased in the pull-down assay, demonstrating the direct binding of PD-L1. **E** qRT-PCR was performed to show quantification of PD-L1 related miRNAs expression after 50 nM DHT treatment. *p < 0.05, NS Not Significant.

Understanding the detailed mechanism of PD-L1 increase due to a high dose DHT is likely to suggest a novel therapeutic strategy to improve BAT. We therefore measured the PD-L1 mRNA expression after a high dose DHT treatment. It was found that the PD-L1 mRNA showed no significant alteration after high dose DHT application (Fig. 3A), indicating a post-transcriptional regulation of PD-L1 expression. To determine whether increased PD-L1 expression was due to enhanced protein stability, the metabolic stability of PD-L1 was measured with the addition of cycloheximide to block the *de novo* protein synthesis. The results showed that a high dose DHT didn’t increase the stability of PD-L1 in EnzS1-C4-2 and EnzR1-C4-2 cells (Fig. 3B). These results led to a logical hypothesis that PD-L1 protein might be regulated at a post-transcriptional level including being regulated by non-coding RNAs (ncRNAs) such as microRNAs (miRNAs). Indeed, Agonaute2 (AGO2) complex pull-down followed by *PD-L1* mRNA detection is consistent with this hypothesis (data not shown), suggesting that miRNAs might be involved in regulating PD-L1 protein expression in response to a high dose DHT treatment.

To identify potential miRNAs that can target 3’UTR of *PD-L1* mRNA, we performed bioinformatic analyses from multiple databases (TargetScan, miRDB, and RNA22), and identified 24 miRNAs with potential linkage to the PD-L1 expression (Fig. 3C). We then applied the RNA pull-down assay to test whether PD-L1 could interact with these candidates. Results revealed that miR-155-5p, miR-378a-3p, miR-513a-5p, miR-138-5p, miR326, miR-141-3p, miR-17-5p, miR-200a-3p, miR-34b-5p, miR-450b-5p and miR-93-5p, but not the other 13 miRNAs, were significantly decreased after DHT treatment in the biotin-oligo pulldown, suggesting a potential direct binding of these 11 candidate miRNAs with PD-L1 mRNA (Fig. 3D). Nevertheless, 4 among the 11 candidates (miR-141-3p, miR-17-5p, miR-200a-3p, miR-93-5p) were excluded because of relative low expression according to qRT-PCR results. Thus, the number of miRNAs candidates was narrowed down to 7. In addition, the expression of these 7 candidates after high dose DHT treatment was also tested, and all appeared to have a significant increase in response to high dose DHT (Fig. 3E), suggesting that additional molecules such as circular RNAs (circRNAs) or linear long non-coding RNAs (lncRNAs) might be involved to negate the effect of increased expression of these miRNAs to result in an increase of PD-L1 protein expression.

### Mechanism dissection of how a high dose DHT/AR can increase the PD-L1 expression: via up-regulating the circFKBP5 expression

**Fig. 4:**
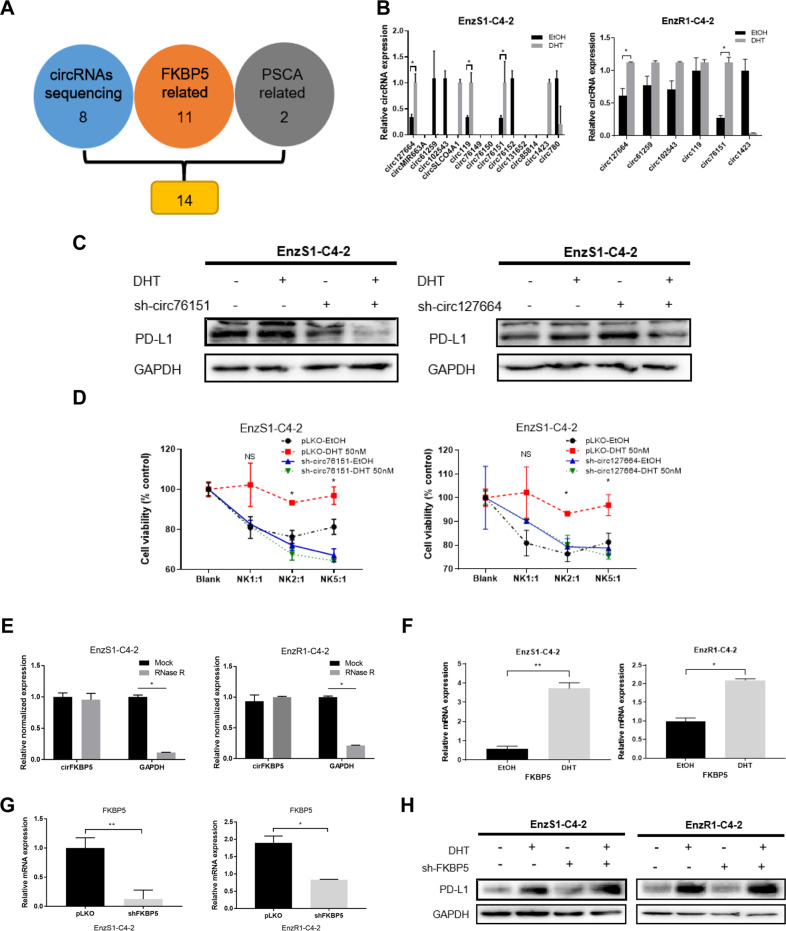
A high dose DHT/AR can increase the PD-L1 expression via up-regulating the circFKBP5 expression. **A** Schematic diagram for prediction of 14 candidate circRNAs that were both related to AR target-genes (FKBP5 and PSCA) **B** qRT-PCR was performed to show quantification of 14 candidate circRNAs expression after 50 nM DHT treatment in EnzS1-C4-2 (left panel) and EnzR1-C4-2 (right panel) cells. **C** Western blot showed PD-L1 expression after sh-circ76151 (left panel), sh-circ127664 (right panel) in both cell lines and treated with 50 nM DHT or EtOH. **D** Before treating EnzS1-C4-2 cells with 50 nM DHT, circ76151 (left panel) and circ127664 (right panel) expressions were knocked down. After treatment, MTT assay was performed separately using NK92-MI cells to target EnzS1-C4-2 cells. **E** RNase R assay to determine the sensitivity of circFKBP5 by RNase R digestion in EnzS1-C4-2 (left panel) and EnzR1-C4-2 (right panel). **F** qRT-PCR was applied to test FKBP5 mRNA expression in EnzS1-C4-2 (left panel) and EnzR1-C4-2 (right panel) after 50 nM DHT treatment for 48 h. **G** qRT-PCR was performed to evaluate the efficacy of knocking down FKBP5 (shFKBP5) in EnzS1-C4-2 (left panel) and EnzR1-C4-2 (right panel) with/without (w/wo) shFKBP5. **H** Western blot showed PD-L1 expression in both cell lines after sh-FKBP5 and treated with 50 nM DHT or EtOH. *p < 0.05 **p < 0.01, NS = Not Significant.

To identify the intermediate molecules that can neutralize the increase of miRNA expression, we decided to focus on circRNAs as they are more metabolically stable, serving as a sponge of miRNAs to activate the expression of miRNA targets [20, 21]. We first collected existing published reports on PCa circRNAs as a source of potential circRNAs that can sponge the likely miRNAs targeting PD-L1. A comprehensive expression and functional profile of circRNAs expression in PC3, CWR22Rv1 and RWPE-1 cell lines led to a conclusion that circRNAs may be closely related to the development and progression of PCa [22]. As a high dose DHT increased PD-L1 expression in an AR-dependent manner, we selected those circRNAs whose expression is positively associated with AR expression in that survey. In addition, we also included those circRNAs from well-known AR target genes, such as FKBP5 (FK506 binding protein 5) and PSCA (prostate stem cell antigen), but there is no circRNA from the canonical AR target gene PSA. Constrained by the predicted miRNAs that can bind to these circRNAs, 14 candidate circRNAs (Fig. 4A) were tested for their expression in response to DHT treatment. The qRT-PCR analysis indicated that two of them, hsa_circ_0076151 and hsa_circ_0127664, showed an increase in expression after treatment with a high dose DHT in both cell lines (Fig. 4B).

In order to further narrow down the circRNA candidates, we suppressed the two circRNAs that were up-regulated by a high dose DHT with shRNAs targeting specifically the junction region of the circRNA. Western blot analysis of the lysates from the EnzS1-C4-2 cells with and without DHT treatment indicated that suppressing both of the two circRNAs could prevent the increase of PD-L1 by a high dose DHT (Fig. 4C). Among them, hsa_circ_0127664 was excluded for further analysis as its suppression unexpectedly led to an increase of PD-L1 expression. We also treated the sh-circRNA-EnzS1-C4-2 cells with NK cells and performed MTT assays (Fig. 4D), the result showed that only knocking down hsa_circ_0076151 was able to reverse the inhibition of NK cytotoxicity due to a high dose DHT, consistent with an increase of PD-L1 expression. Therefore, we focused on studying the hsa_circ_0076151, which is generated from the sequence within the exons of FKBP5, and renamed it as circFKBP5. Digestion with the RNase R further validated the circular nature of this ncRNA as RNase R failed to decrease its level while it can readily decrease the linear mRNA of *GAPDH* (Fig. 4E). To exclude the possibility that shRNA targeting circFKBP5 blocks the increase of PD-L1 through non-specific targeting of linear FKBP5 mRNA, we also directly targeted linear *FKBP5* mRNA outside the region of this circRNA, and the results indicated that linear FKBP5 was induced by a high dose DHT (Fig. 4F), however its suppression by shRNA (Fig. 4G) did not lead to reverse the impact on PD-L1 expression by DHT (Fig. 4H), solidifying the role of circFKBP5 in this process.

Together, results from Fig. 4A–H suggest that a high dose DHT/AR signals may function via up-regulating circFKBP5 to increase PD-L1 expression, and subsequently inhibit the NK cell cytotoxicity.

### Mechanism dissection of how a high dose DHT/AR/circFKBP5 axis can increase PD-L1 expression: via sponging miR-513a-5p expression

**Fig. 5:**
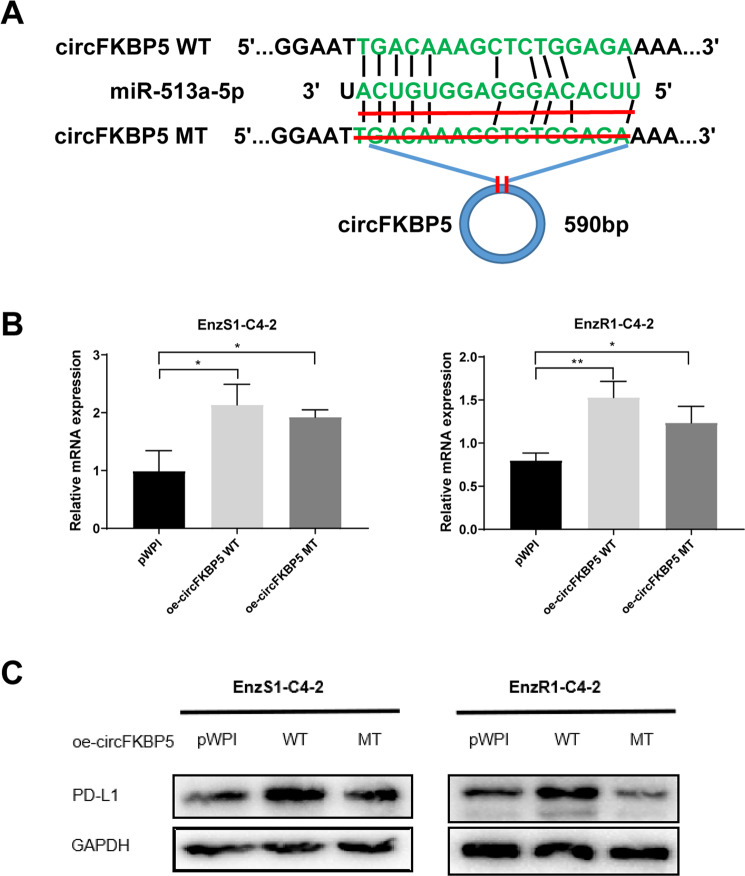
A high dose DHT/AR/circFKBP5 axis can increase PD-L1 expression via sponging miR-513a-5p expression. **A** The circFKBP5 with and without miR-513a-5p binding sites (CircNet). **B** qRT-PCR was performed to show quantification of circFKBP5 after oe-mutant circFKBP5 in EnzS1-C4-2 (left panel) and EnzR1-C4-2 (right panel) cells. **C** WB showed that, compared to wild type, oe-mutant circFKBP5 failed to increase PD-L1 expression significantly in CRPC cells. *p < 0.05, **p < 0.01.

Based on the bioinformatic analysis, the functional characterization of circFKBP5 led to the identification of miR-513a-5p as a likely candidate that targets PD-L1 expression. To directly test whether miR-513a-5p mediates the regulation of PD-L1 expression by circFKBP5, we generated a mutant circFKBP5 deficient in binding to this miRNA (Fig. 5A). Expression of both wild type and mutant circFKBP5 in both cell lines were roughly comparable (Fig. 5B), however mutant circFKBP5 failed to increase PD-L1 expression as compared to the wild type circFKBP5 in both EnzS1-C4-2 and EnzR1-C4-2 cells (Fig. 5C). These results strongly support the conclusion that circFKBP5 sponges the miR-513a-5p to increase the PD-L1 expression in response to a high dose DHT treatment.

Together, results from Fig. 5A–C indicated that high dose DHT/AR/circFKBP5 axis can increase PD-L1 expression through sponging the miR-513a-5p expression.

### Mechanism dissection of how high dose DHT/AR/circFKBP5/miR-513a-5p axis can increase the PD-L1 expression: via binding to the 3’UTR of PD-L1 mRNA

**Fig. 6:**
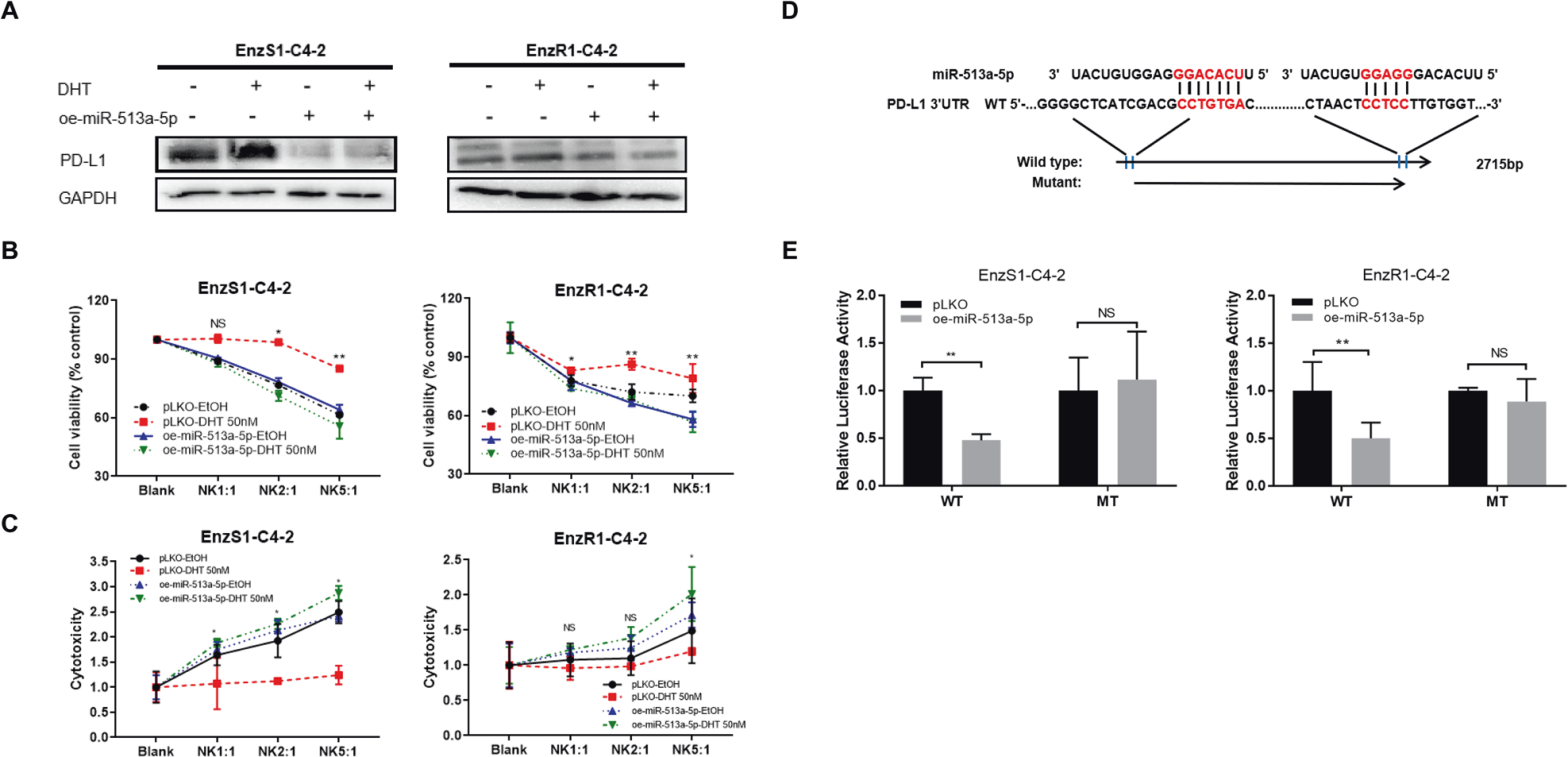
miR-513a-5p can regulate PD-L1 via binding to the 3’UTR of PD-L1 mRNA. **A** Western blot of oe-miR-513a-5p in both cell lines showed PD-L1 expression alternation after treatment with 50 nM DHT or EtOH. **B**, **C** Before treating EnzS1-C4-2 (left panel) and EnzR1-C4-2 cells (right panel) with 50 nM DHT or EtOH, miR-513a-5p expression was over-expressed. After treatment, MTT (**B**) and LDH **C** assays were performed separately using NK92-MI cells to target CRPC cells. **D** Sequence alignment of the PD-L1 3’UTR with wild type (WT) and potential mutant (MT) miR-513a-5p targeting sites. **E** Luciferase reporter activity after transfection of wild type or mutant PD-L1 3’UTR reporter construct in EnzS1-C4-2 (left panel) and EnzR1-C4-2 cells (right panel) with/without oe-miR-149-3p.*p < 0.05 **p < 0.0, NS Not Significant.

To confirm whether miR-513a-5p indeed plays a vital role in the pathway, we overexpressed miR-513a-5p, which was significantly decreased in the biotin-oligo pulldown after high dose DHT treatment (See Fig. 3E). The results indicated that overexpressing miR-513a-5p could inhibit the increase of PD-L1 resulting from DHT in both cell lines (Fig. 6A). The decreased NK cytotoxicity towards CRPC cells by DHT was also reversed after miR-513a-5p overexpression (Fig. 6B, C). To determine that high dose DHT/AR/circFKBP5/miR-513a-5p axis can affect the PD-L1 expression via binding to the 3’UTR of PD-L1 mRNA, we identified potential miRNA binding sites (http://targetscan.org/) with subsequent construction of the reporter plasmids using the psicheck2 vector carrying the wild type (WT) and mutant miRNA-target sites (Fig. 6D). As expected, the luciferase assay results revealed that overexpressing miR-513a-5p markedly decreased luciferase activity in EnzS1-C4-2 and EnzR1-C4-2 cells transfected with wild type (WT) PD-L1 3’UTR but not the mutant (MT) PD-L1 3’UTR (Fig. 6E).

Together, results from Fig. 6A–E suggest that a high dose DHT/AR/circFKBP5/miR-513a-5p axis can increase the PD-L1 expression via circFKBP5 sponging miR-513a-5p binding to the 3’UTR of PD-L1 mRNA.

### Preclinical study using in vivo mouse model to test whether PD-L1-shRNA can increase the efficacy of high dose DHT to better inhibit CRPC cell growth

**Fig. 7:**
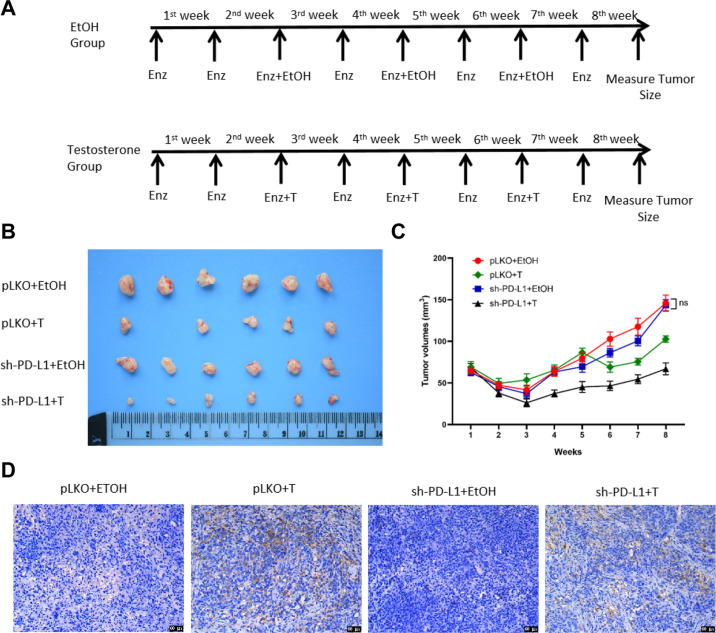
A lower PD-L1 expression can enhance high dose androgen’s suppression of tumor growth in vivo. **A** Treatment schecule of nude mice groups injected with EnzR1-C4-2 cells transfected with pLKO or sh-PD-L1 at suspension of 5 × 10^6^ cells. Mice were injected with Enz (10 mg/kg, twice weekly). Bipolar therapy treatment was conducted by injection of Testosterone (200 μg/kg, twice weekly) or EtOH (Ethanol) during the 3rd/5th/7th week. Mice were sacrificed after 8 weeks, tumors were removed and measured for studies. **B**, **C** Xenograft tumors from mice after eight weeks’ drug treatment are shown. Knocking down PD-L1 **B** increases high dose androgen sensitivity and decreases xenograft tumor size. Tumor sizes were measured every week to calculate tumor volumes (**C**), with reduction of the xenografts observed in sh-PD-L1 plus Testosterone treatment group. **D** Representative IHC images of PD-L1 expression in tumor tissue samples from the four groups, 200× magnification. NS Not Significant.

To test whether suppression of PD-L1 can enhance the killing effect of NK cells and further inhibit PCa tumor growth under BAT in vivo, we generated EnzR1-C4-2 cells with stable expression of sh-PD-L1 or vector control (pLKO) cells and subcutaneously implanted these cells into nude mice for 4 treatment groups for each cell line as follows, 1: pLKO+EtOH; 2: pLKO+Testosterone; 3: sh-PD-L1 + EtOH; and 4: sh-PD-L1 + Testosterone (Fig. 7A). The subcutaneous tumor growth was monitored/measured for 8 weeks by caliper then mice were sacrificed and tumors removed for studies. We analyzed the data and found that the combination treatment of sh-PD-L1 plus Testosterone could significantly decrease tumor growth **(**Fig. 7B**)** and achieve greater tumor growth suppression **(**Fig. 7C**)** than treatment with either sh-PD-L1 or Testosterone alone. To detect PD-L1 in each group of collected subcutaneous tumor samples we used immunostaining. PD-L1 stainings were significantly enhanced in Testosterone alone group and sh-PD-L1 plus Testosterone group compared with vehicle group (Fig. 7D) which is consistent with our in vitro results.

**Fig. 8:**
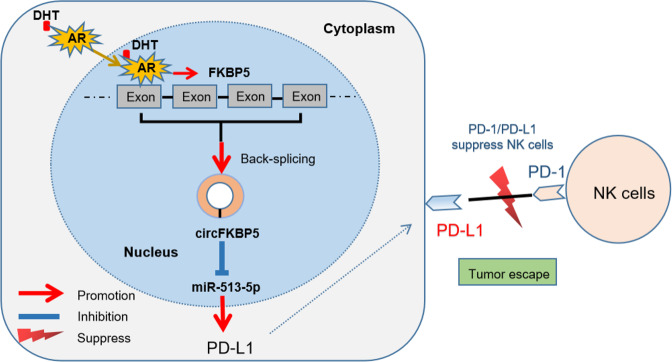
A mechanistic diagram. A mechanistic diagram for DHT suppression effect on NK cells immunotherapy efficacy towards CRPC cells.

Together, in vivo results show that a lower PD-L1 expression will enhance the inhibitory effect of high dose androgen treatment (Fig. 8).

## Discussion

NK cells are lymphocytes of innate immunity with cytotoxicity against cancer cells [23]. They participate in the immune response against cancers due to their ability to recognize molecular characteristics of stressed tumor cells, such as missing self or inducing self-recognition [19]. The presence of NK cells in tumor microenvironment often means a favorable clinical outcome [24, 25]. It has been widely accepted, also in PCa, that the PD-L1/PD-1 axis plays a crucial role in inhibiting cytotoxic T cells and maintaining an immunosuppressive cancer microenvironment [26]. Accordingly, PD-L1/PD-1 checkpoint blockade immunotherapy unleashes CD8^+^ T cells to kill cancer cells. However, Hsu et al used several mouse models and found that PD-1 was also expressed on NK cells, and PD-L1 expression on tumor cells led to impaired NK cell immune effect, thus the generation of more aggressive tumors [19]. Several studies demonstrated that apart from T cells, NK cells could also mediate the PD-L1/PD-1 blockade effect and are fundamental for the full therapeutic effect of immunotherapy [19, 27, 28], in a manner without requiring cancer cells expression of neoantigens or upregulation of self-antigens [29]. In our study, we found a high dose DHT could increase the PD-L1 expression in EnzS1-C4-2 and EnzR1-C4-2 cells, which weakened the susceptibility of these CRPC cells to NK cell cytotoxicity, supporting a role of PD-L1 mediated innate immune surveillance by NK cells.

ADT has been well recognized to be effective to inhibit PCa progression and widely used in clinics for several decades. In recent years, BAT, which seems to contradict ADT on its surface, has shown its ability to suppress PCa growth in the preclinical research with xenograft models and human PCa cell lines [30–32]. With the cycling of androgen deprivation and high dose androgen activation, the efficacy of BAT has further been confirmed in selected patients by pilot clinical trials [9, 33]. On the other hand, the emergence of PD-L1/PD-1 inhibitors likely is the greatest progress in the treatment of advanced cancers during the last few decades. To date, there have been several published papers [34–36] and ongoing clinical trials involving the use of PD-L1/PD-1 inhibitors in PCa patients. Now it has been generally accepted that these inhibitors do not show obvious effect in some patients. Those potential subsets of PCa patients who derive significant benefits from checkpoint inhibitors are usually men with tumors that have high expression of PD-L1 [37] and/or a high mutational burden [38]. In this general context, we examined a potential connection between BAT and immune checkpoint therapy. We used Enz-sensitive and Enz-resistant CRPC cells to evaluate the efficacy of high dose DHT in light of the participation of immune surveillance. We found that lower doses of DHT (1 nM and 10 nM) could not inhibit NK cells cytotoxicity, but high dose DHT (50 nM) could, thus contributing to immune escape from NK cells. Furthermore, we found this NK escape is due at least in part to the increased expression of PD-L1. Indeed, our in vitro and in vivo studies indicated that targeting PD-L1 with sh-RNA or anti-PD-L1 antibody could revive the susceptibility of CRPC to NK cell cytotoxicity after a high dose DHT treatment. These findings help to explain the partial effectiveness of BAT in select patients, potentially as a result of resistance to inhibition of immune participation, while raising the possibility of one novel means to boost the efficacy of BAT with a combination of PD-L1/PD-1 checkpoint blockade therapy. Thus, for Enz-resistant CRPC, the combination therapy of BAT and anti-PD-L1 may be a novel therapeutic approach that deserves further investigation.

Our mechanism dissection revealed that a high dose DHT/AR might regulate PD-L1 expression via circFKBP5/miR-513a-5p/PD-L1 signaling. Our results support the view that circRNAs, as non-coding RNAs, could play a crucial role in the development and progression of PCa [22, 39], and could be potential therapeutic targets for PCa [40]. The well-known important functional mechanism of circRNAs is sponging miRNAs like competitive endogeneous RNA molecules [41]. The miRNAs could inhibit gene expressions by binding to the 3’UTR of their target mRNAs [42]. Previously, the miRNAs were reported to be vital regulators of biologic processes in PCa progression, which might constitute valuable biomarkers for the diagnosis, prognosis, and therapeutic options in PCa patients [43, 44]. Coincidentally, miR-513a-5p has been shown to possess oncogenic potential in breast cancer [45] and glioblastoma [42], while in contrast act as a tumor suppressor gene in osteosarcoma tissues [46]. In our research, up-regulation of circFKBP5 due to DHT/AR acted as a miR-513a-5p sponge, thereby negated miR-513a-5p’s inhibitory effect on its target gene PD-L1 by binding to the 3’UTR of PD-L1 mRNA, exemplifying the so-called circRNA-miRNA-mRNA network [22, 39].

In summary, our study uncovered a novel mechanism for how a high dose DHT treatment inhibits susceptibility of CRPC cells to NK cell cytotoxicity via AR/circFKBP5/miR-513a-5p/PD-L1 signals. This finding might help in the development of potential new therapeutic choices, such as anti-PD-L1 therapy, to reinforce BAT efficacy for patients.

## Supplementary information

aj-checklist

Full length western blots

## Data Availability

The data used and/or analyzed during the current study are available from the corresponding author on reasonable request.
