# Supplementary material for: High dose androgen suppresses natural killer cytotoxicity of castration-resistant prostate cancer cells via altering AR/circFKBP5/miRNA-513a-5p/PD-L1 signals
Source: Cell Death Dis. 2022 Aug 29;13(8):746. doi: 10.1038/s41419-022-04956-w (PMC9424293; doi:10.1038/s41419-022-04956-w)

Figure 1D shAR

C4-2

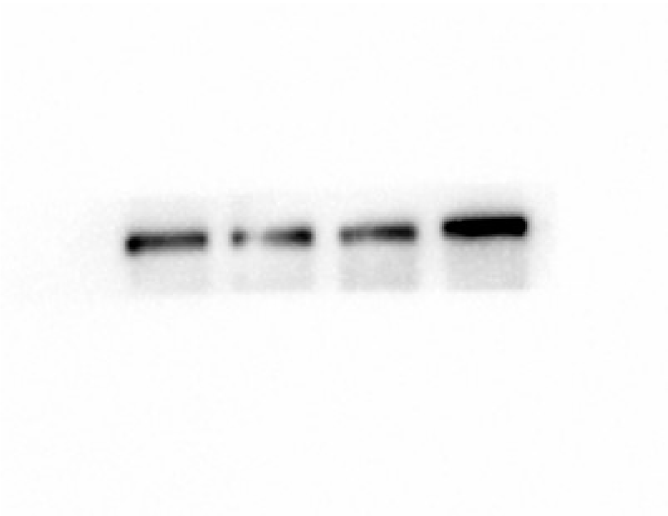

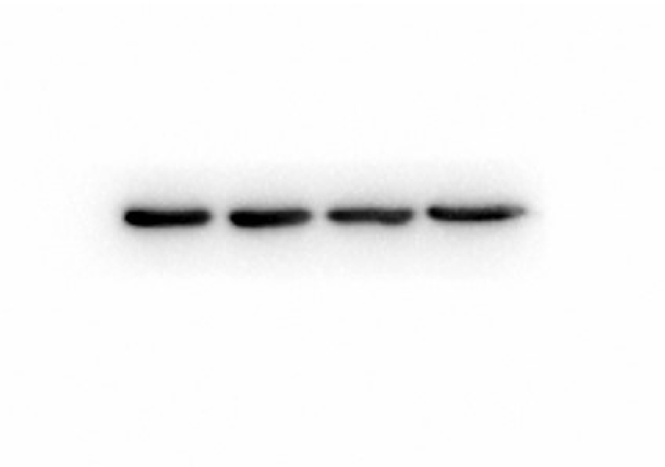

C4-2R

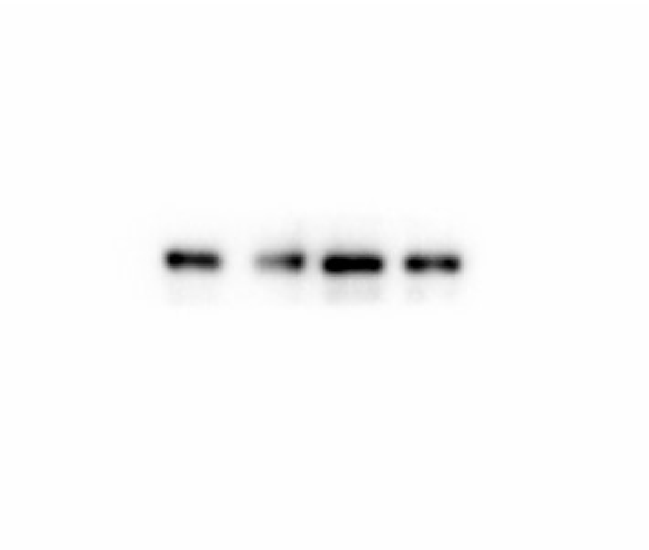

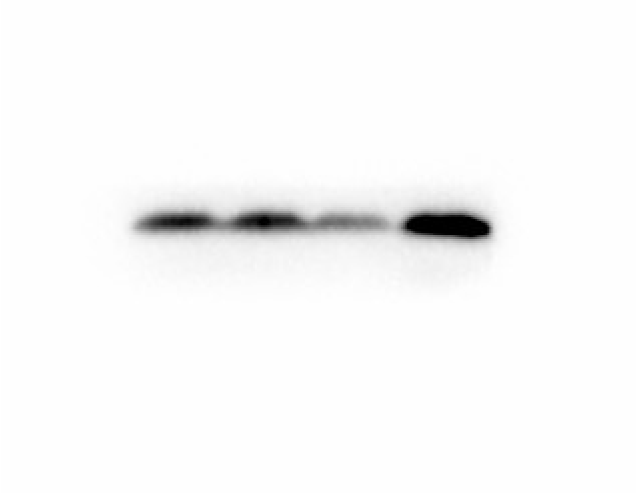

Figure 2A DHT 48h PD-L1

C4-2

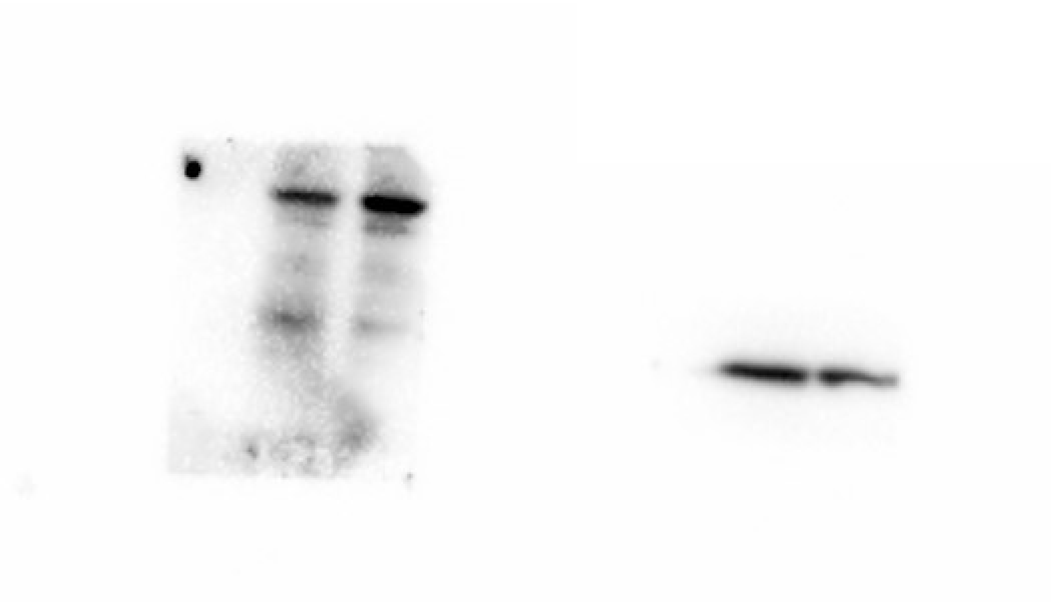

C4-2R

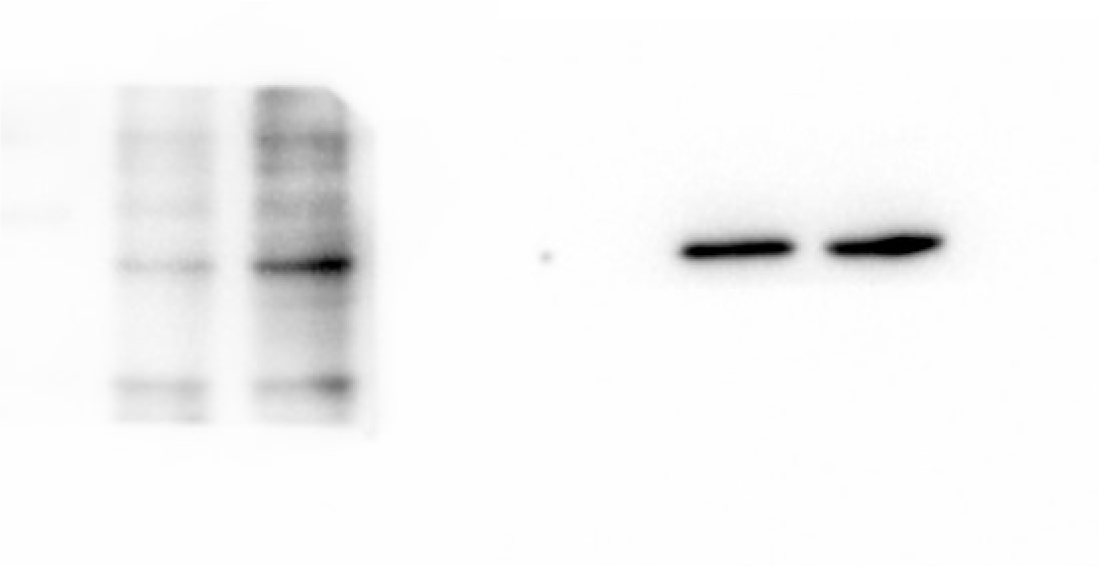

Figure 2B DHT 48h+36h PD-L1

C4-2

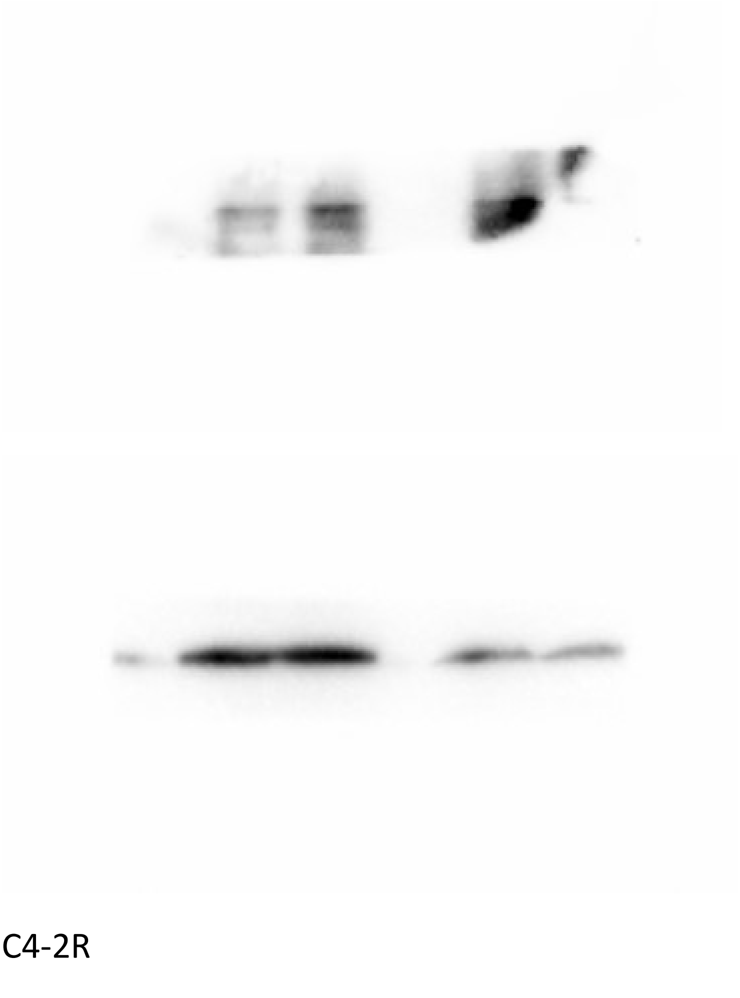

C4-2R

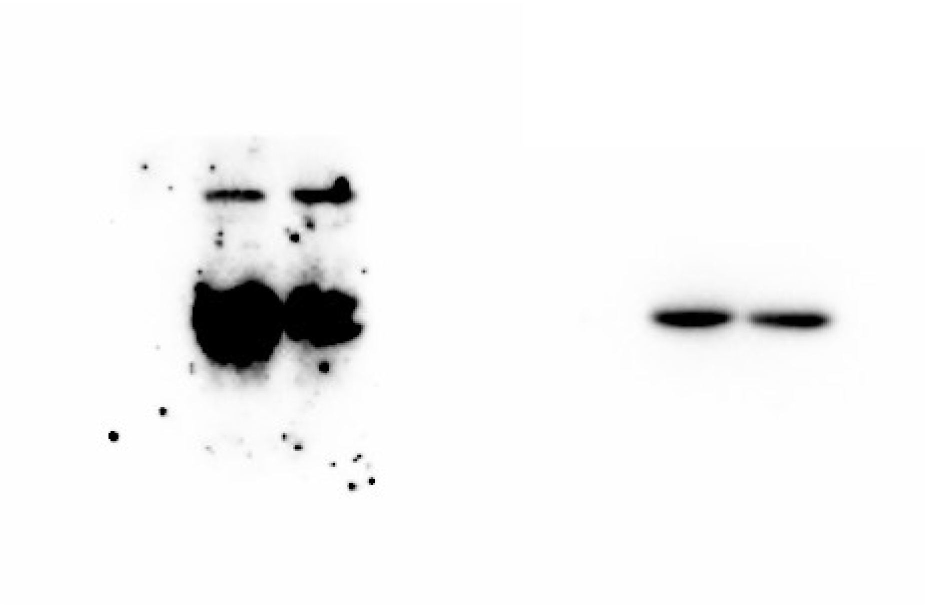

Figure 2C DHT shAR PD-L1

C4-2

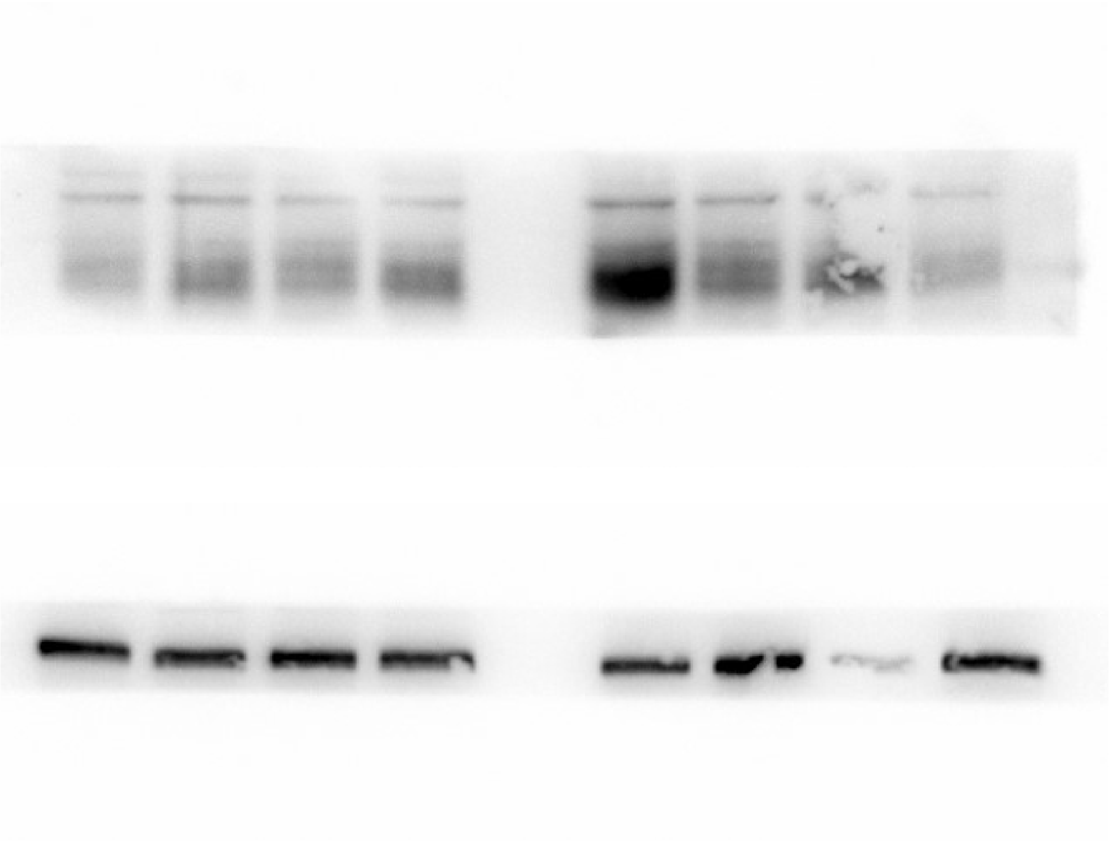

C4-2R

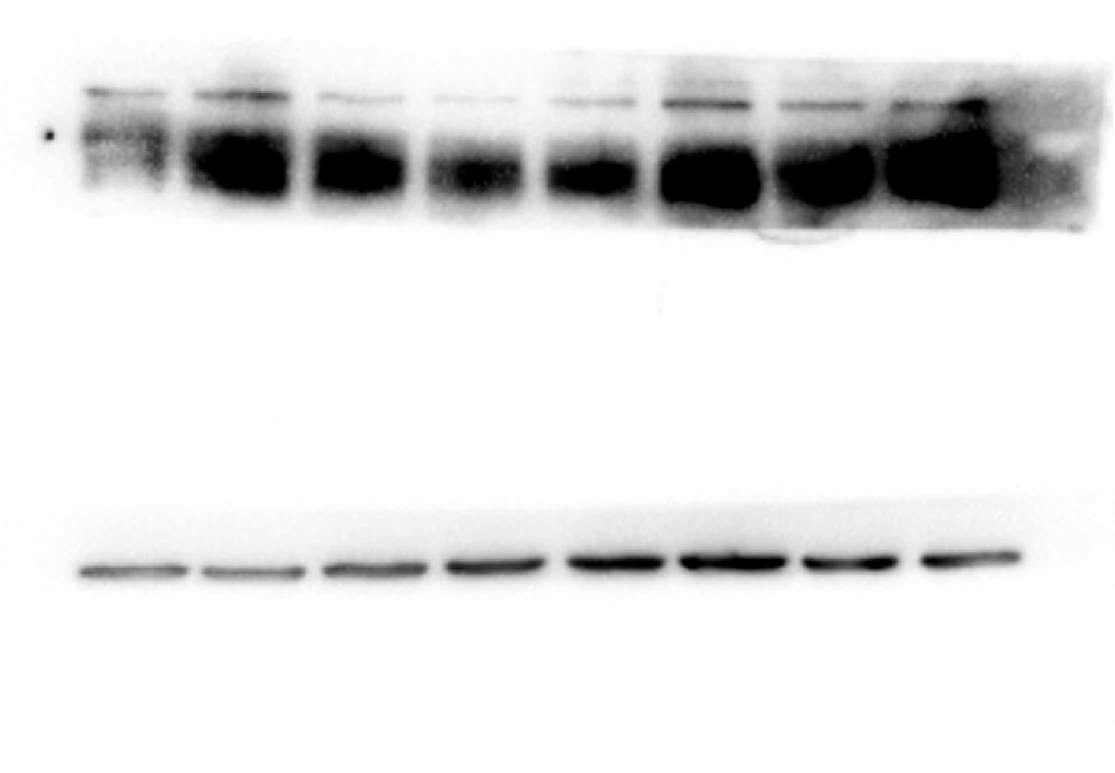

Figure 3B DHT PD-L1 protein stability

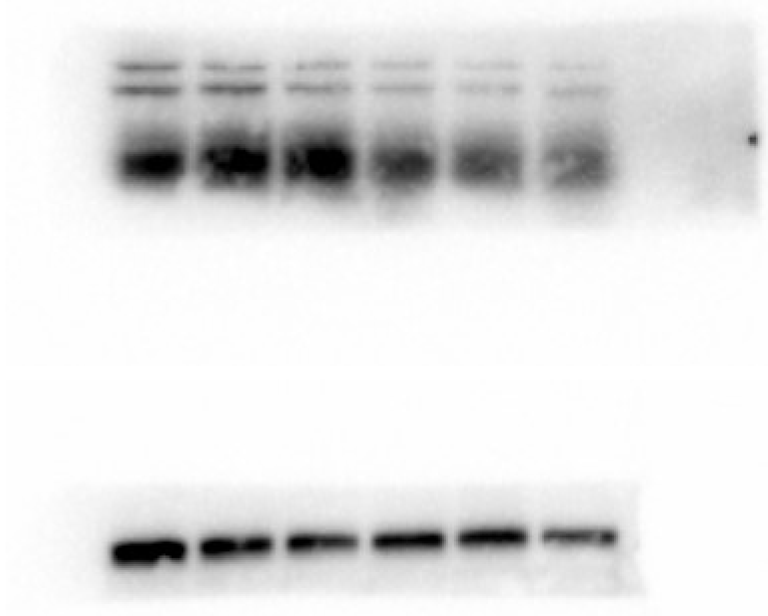

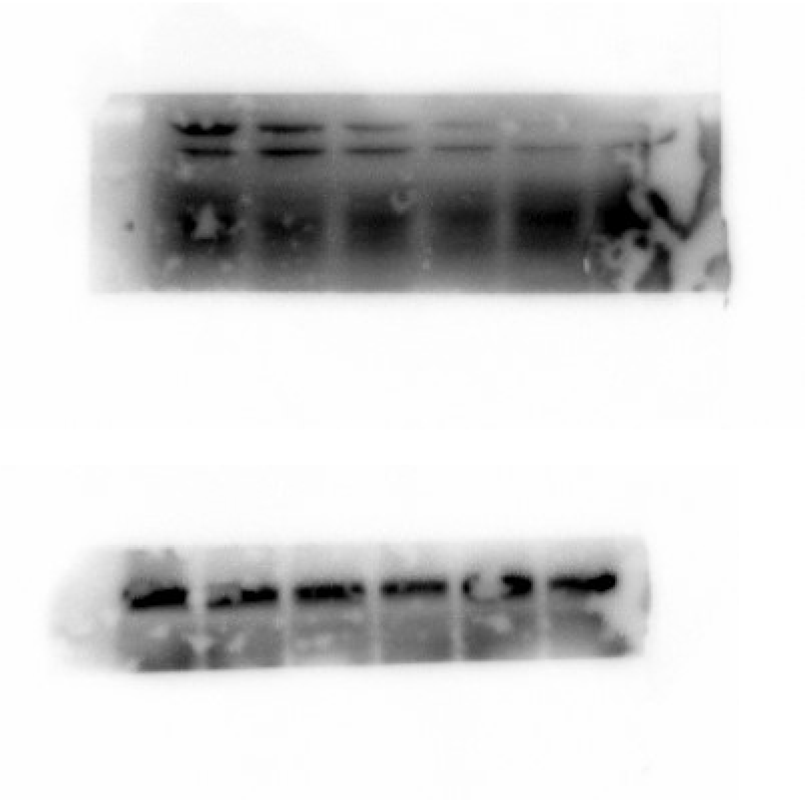

Figure 4C DHT sh-circRNA PD-L1

sh76151 PDL1

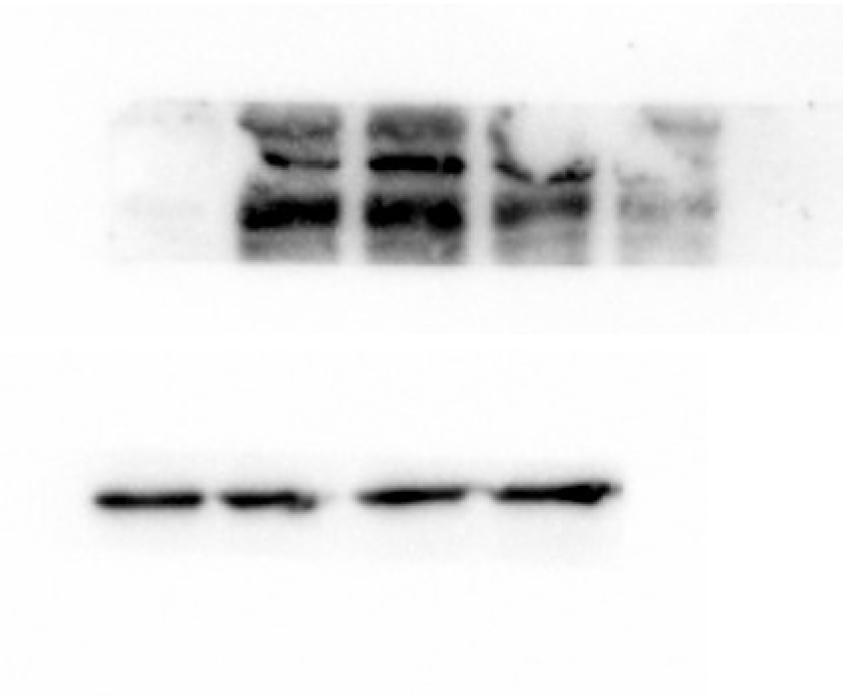

sh127664 PDL1

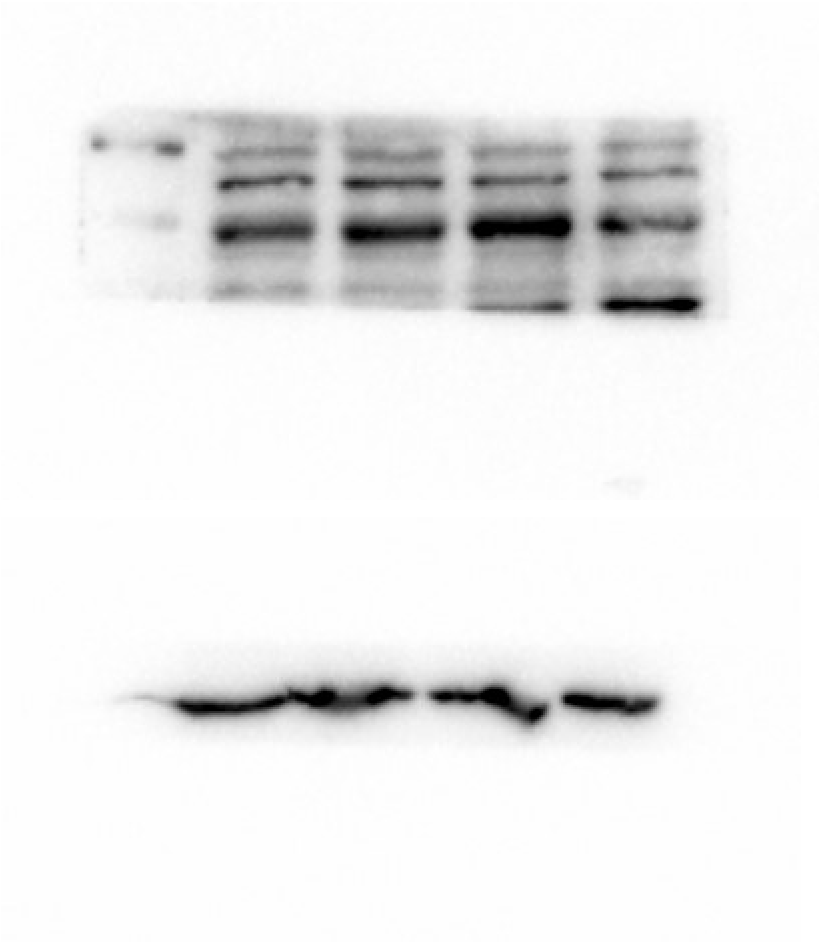

Figure 4I DHT shFKBP5 PD-L1

C4-2

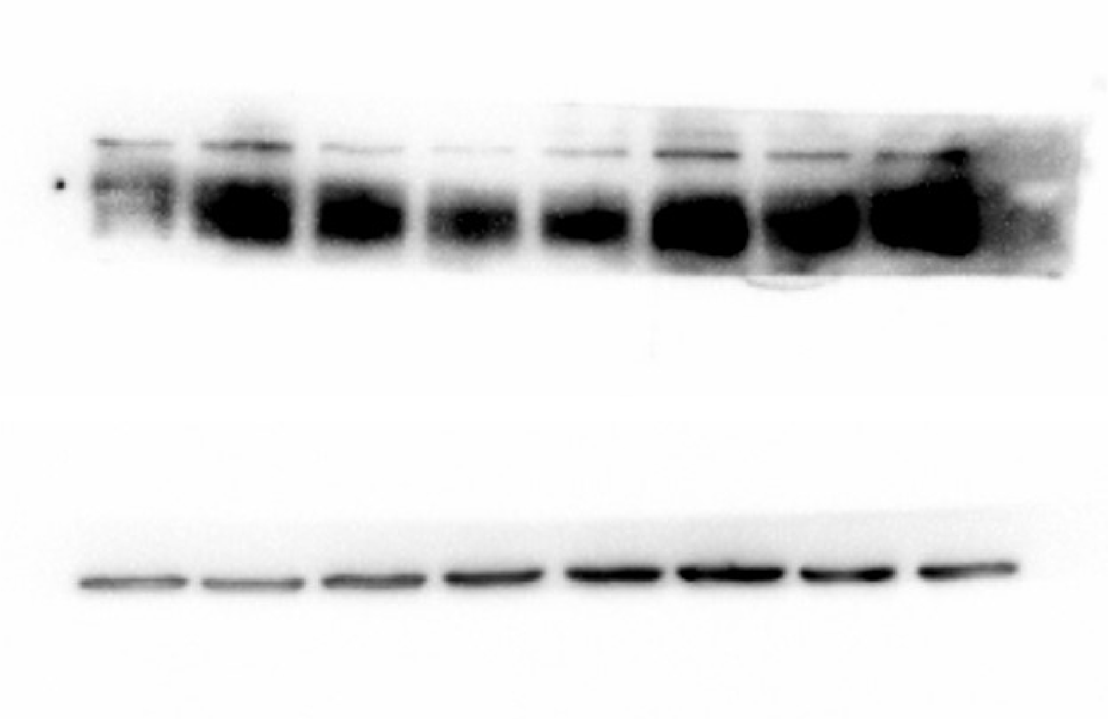

C4-2R

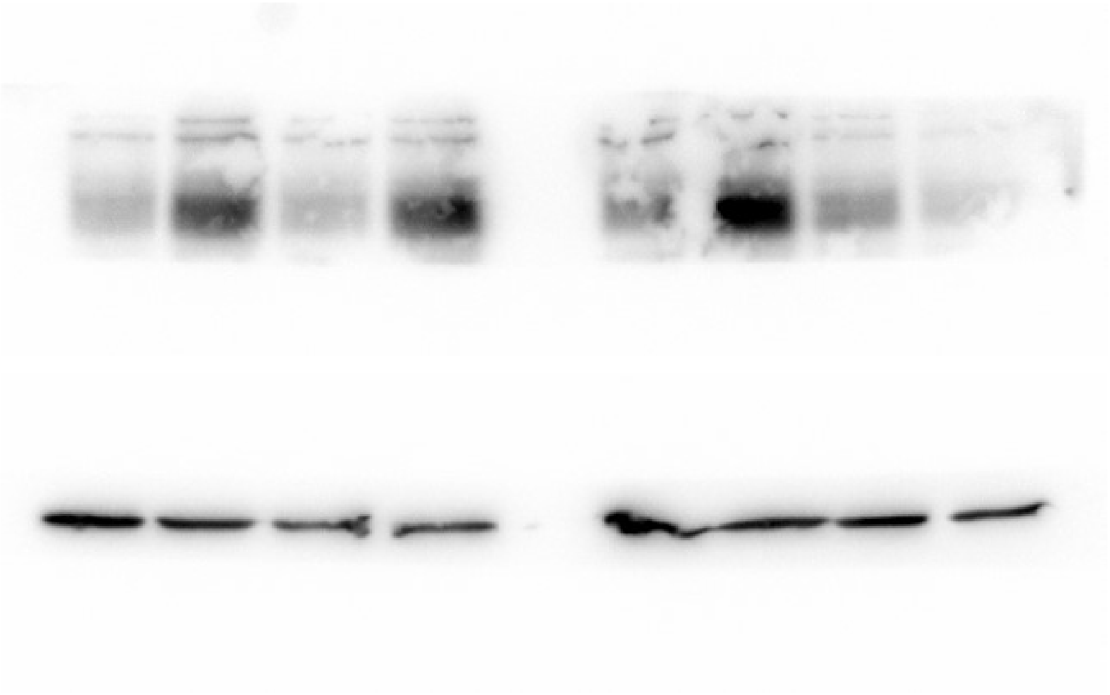

Figure 5C oe-circRNA WT MUT PD-L1

C4-2

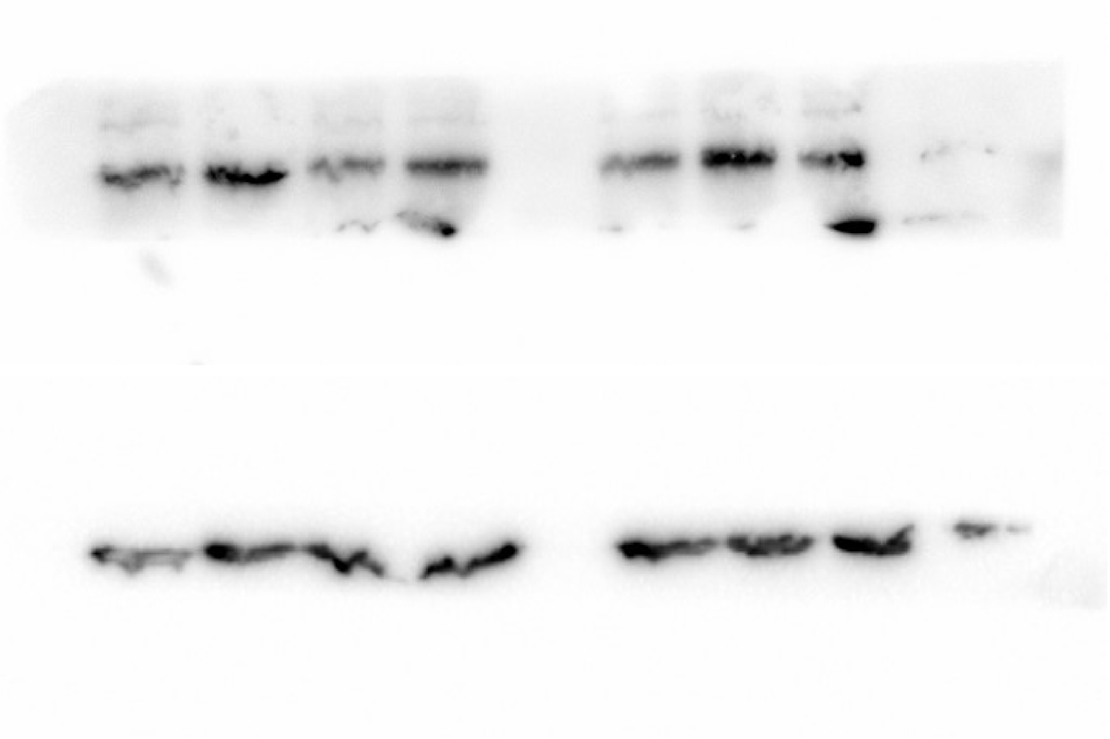

C4-2R

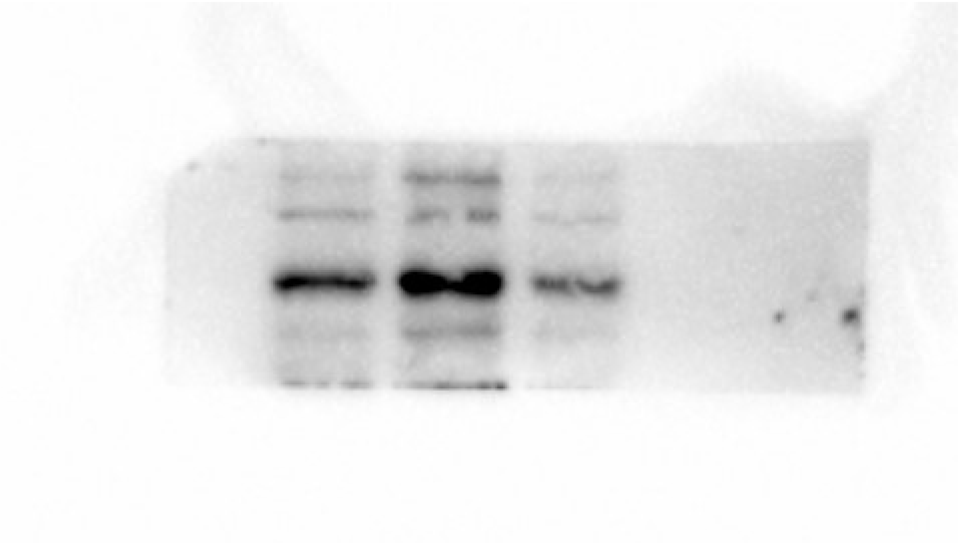

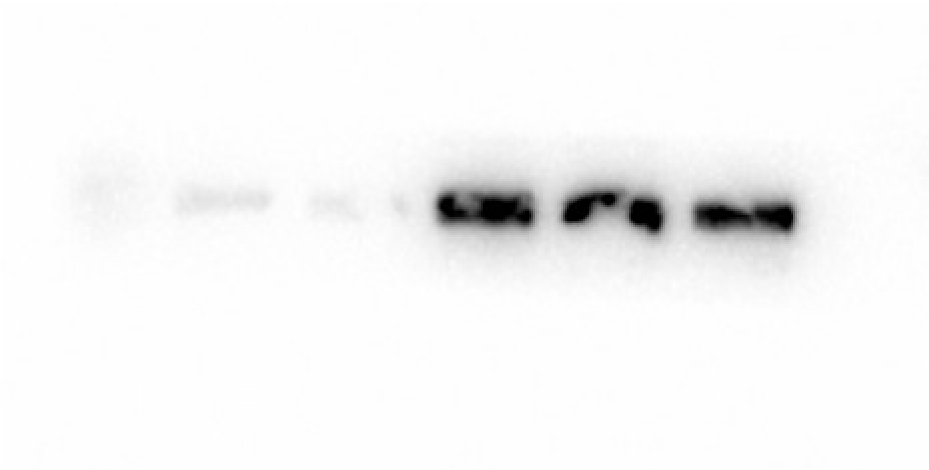

Figure 6A DHT oe-miRNA PD-L1

C4-2

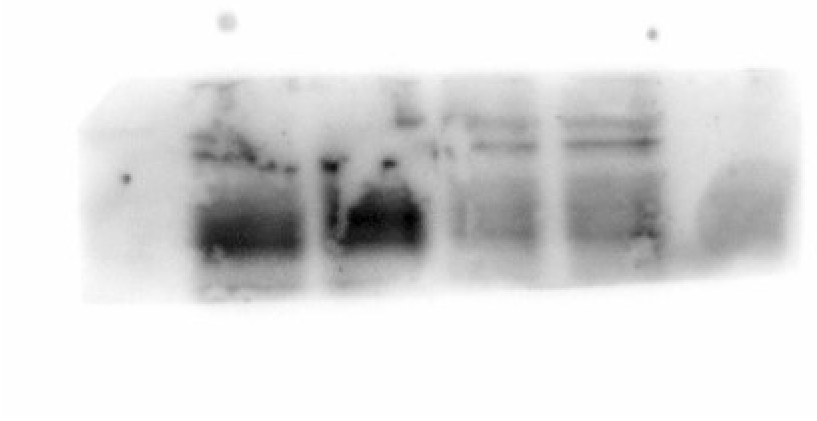

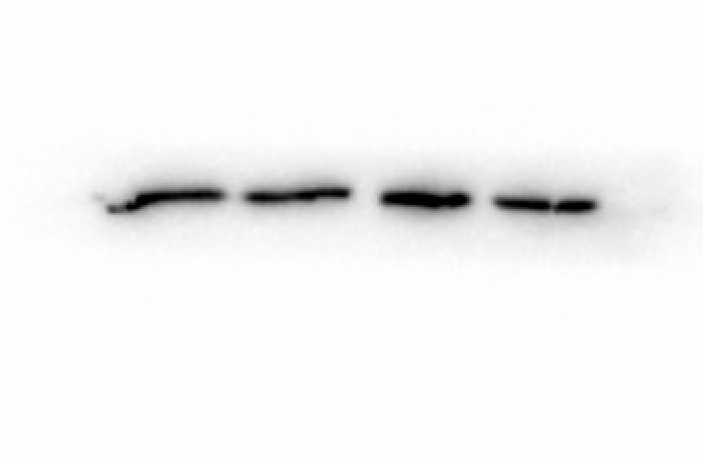

C4-2R

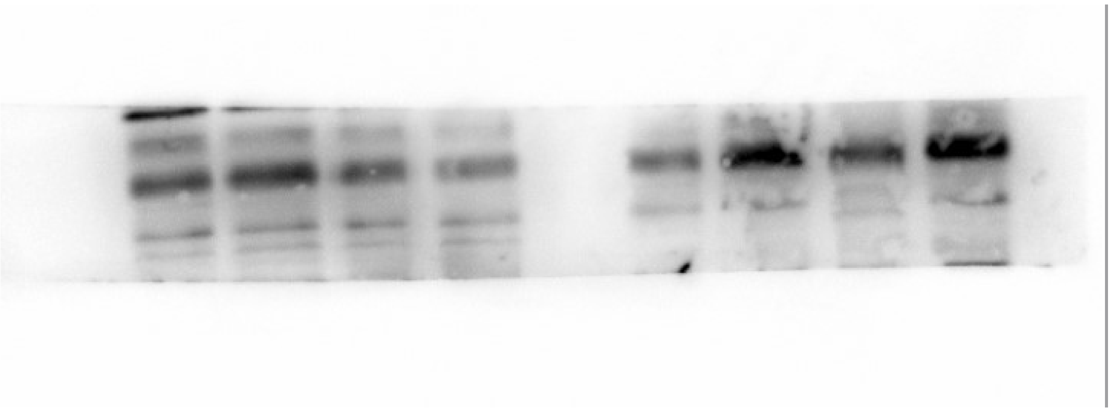

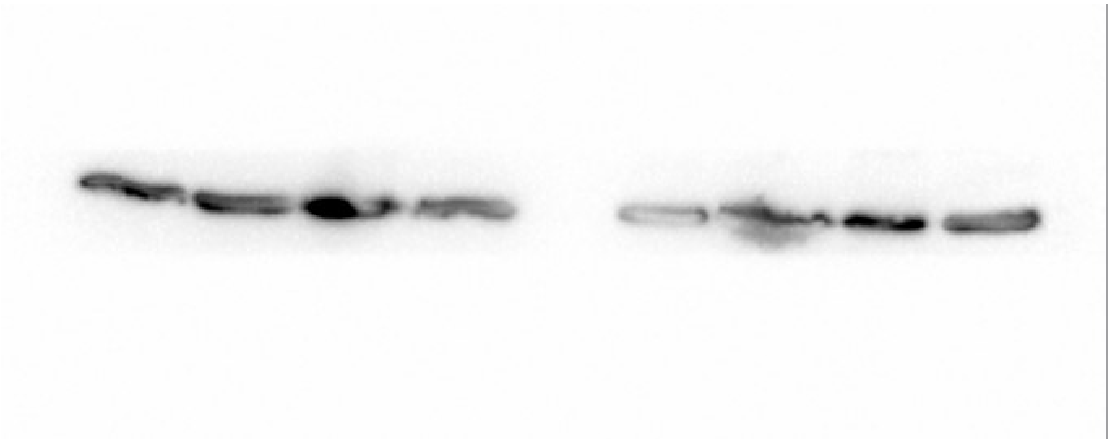

Supplement: Supplementary file 2 — Full length western blots [file 41419_2022_4956_MOESM2_ESM.pdf]
